# Nuclear Receptors and Clock Components in Cardiovascular Diseases

**DOI:** 10.3390/ijms22189721

**Published:** 2021-09-08

**Authors:** Benoit Pourcet, Hélène Duez

**Affiliations:** Univ. Lille, Inserm, CHU Lille, Institut Pasteur de Lille, U1011-EGID, F-59000 Lille, France

**Keywords:** atherosclerosis, cardiovascular diseases, circadian rhythm, chronobiology, nuclear receptors, Rev-erb, ROR, inflammation

## Abstract

Cardiovascular diseases (CVD) are still the first cause of death worldwide. Their main origin is the development of atherosclerotic plaque, which consists in the accumulation of lipids and inflammatory leucocytes within the vascular wall of large vessels. Beyond dyslipidemia, diabetes, obesity, hypertension and smoking, the alteration of circadian rhythms, in shift workers for instance, has recently been recognized as an additional risk factor. Accordingly, targeting a pro-atherogenic pathway at the right time window, namely chronotherapy, has proven its efficiency in reducing plaque progression without affecting healthy tissues in mice, thus providing the rationale of such an approach to treat CVD and to reduce drug side effects. Nuclear receptors are transcriptional factors involved in the control of many physiological processes. Among them, Rev-erbs and RORs control metabolic homeostasis, inflammatory processes and the biological clock. In this review, we discuss the opportunity to dampen atherosclerosis progression by targeting such ligand-activated core clock components in a (chrono-)therapeutic approach in order to treat CVD.

## 1. Introduction

In 2016, 17.9 million people died from CVD, which is 31% of deaths worldwide, with more than three out of four occurring in low- and mid-income countries. Despite a huge effort to prevent and treat CVDs, they are still the first cause of death worldwide (WHO and [1]). Strikingly, because 85% of these deaths are due to ischemic heart diseases and stroke, according to the WHO, atherosclerosis may be seen as the primary cause of CVDs, even if some strokes may result from events independent of plaque rupture.

**Figure 1:**
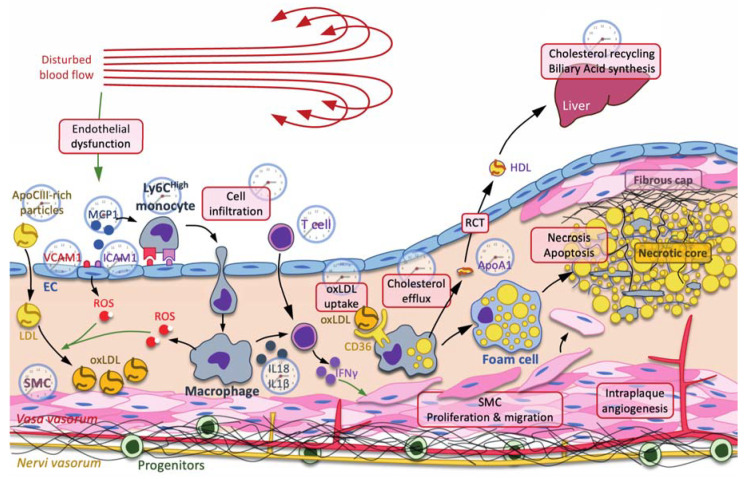
The clock and atherogenesis. Atherosclerosis is triggered by small dense low-density lipoproteins (LDL) and ApoC-III-rich remnant particles that accumulate in regions where the blow flow is disturbed. Low shear stress usually induces a mechanic stress, promoting endothelial dysfunction characterized by an increase in reactive oxygen species (ROS) production, secretion of cytokines, including monocyte chemoattractant protein 1 (MCP1), production of adhesion molecules, including vascular cell adhesion molecule 1 (VCAM1) and intercellular adhesion molecule 1 (ICAM1), and increased permeability to LDL and immune cells. Immune cells, such as neutrophils, monocyte-derived macrophages, T cells, etc., are then recruited to the subendothelial space. Infiltrated LDL are oxidized by reactive oxygen species (ROS) produced by endothelial cells (ECs) and macrophages. Oxidized LDL (oxLDL) are preferentially taken up by macrophages through the scavenger receptor CD36 for cholesterol recycling through the reverse cholesterol transport (RCT). In this pathway, ATP-binding cassette ABCA1 and ABCG1 mediate the efflux of cholesterol from the macrophage to ApoA1 and preβ-high-density lipoprotein (HDL). Cholesterol is transported back to the liver by HDL, where it is recycled in other lipoproteins or used as substrate in the biliary acid biosynthetic pathway. When RCT is insufficient and macrophages overwhelmed by massive cholesterol uptake, macrophages eventually become foam cells, where the accumulation of lipids leads to necrosis and apoptosis, thus forming the necrotic core. In addition to cholesterol metabolism, macrophages also produce cytokines, such as NLRP3 inflammasome-processed IL-18 and IL-1β interleukins, which, together with IL-12, activate the T cell-dependent production of interferon (IFN) γ, among others. IFNγ stimulates the proliferation and the migration of smooth muscle cells (SMC) toward the necrotic core in order to stabilize it by secreting fibers, including collagen, thus forming the fibrous cap. Each process or molecule that is regulated by one or several clock components is represented by a clock. Green arrow: induction.

Atherosclerosis is now commonly defined as a chronic inflammatory disease of the vascular wall consisting in the internalization of lipids, mainly small dense LDL and VLDL-remnants [1], that trigger the recruitment of leucocytes, including neutrophils, monocytes/macrophages, dendritic cells, T cells, B cells and mastocytes [2,3] (Figure 1). Lipids usually accumulate in the vasculature in areas where the blood flow is disturbed [4,5], thus facilitating endothelium damage and promoting endothelial dysfunction [6,7]. Endothelial cell impairment promotes LDL internalization, leucocyte recruitment and the oxidation of LDL [8], which are preferentially taken up by recruited macrophages, becoming foam cells (Figure 1). Foam cells then die by apoptosis and necrosis, releasing their lipid and cellular content, thereby contributing to the formation of the necrotic core [9]. In addition, macrophages produce both cytokines that activate T cells and subsequent interferon γ, which induces the proliferation and migration of smooth muscle cells toward the subendothelial area in order to form a fibrous cap that stabilizes the necrotic core (Figure 1). However, as macrophages produce matrix metalloproteases, this fibrous cap becomes thinner, thus destabilizing the plaque, which becomes prone to rupture [10]. In addition, processes involved in plaque complexification, including intimal calcification and neovascularization, are also important events that promote plaque rupture, thrombus formation and acute vascular events [11,12] (Figure 1).

Although atherogenesis is a natural process of vascular aging, many environmental and genetic factors, such as diabetes, familial hypercholesterolemia, hypertension, smoking, a sedentary lifestyle and obesity, were identified as important risk factors that accelerate this pathogenic process. Interestingly, physiological parameters of the cardiovascular system, including blood pressure and heart rate, display circadian patterns [13]. If the maintenance of a day–night difference in blood pressure is a feature of a healthy cardiovascular system, clinical studies demonstrated diurnal variations in cardiovascular events. For instance, the thromboembolic event frequency peaks during the morning to noon period in humans [14], explaining the higher rate of non-fatal myocardial infarction, infarct size increase and sudden cardiac death during this period [15,16,17]. Numerous studies have also reported that clock disruption in shift workers increases cardiovascular risk factors, including hypertension, diabetes and dyslipidemia. Accordingly, numerous pro-atherogenic factors, including plasma lipid levels and endothelial function, display diurnal variations independently of food intake. Additionally, levels of circulating and infiltrated leucocytes, as well as the activity of the immune system (cytokine secretion, pathogen response and phagocytosis [18]), exhibit diurnal oscillations. Although these studies did not show a direct relationship between clock disruption and atherogenesis, it was later shown that shift work and acute circadian misalignment were associated with subclinical atherosclerosis, measured as higher intima-media thickness and an elevated systemic inflammation, even after adjusting for age and common risk factors [19,20,21,22]. Finally, a lower sleep duration and fragmented sleep are independently associated with an increased risk of subclinical coronary and non-coronary atherosclerosis [23]. The alteration of circadian rhythms is then recognized as a new risk factor facilitating the development of atherosclerotic plaque and acute coronary syndrome. At the molecular level, circadian oscillations in clock gene expression are attenuated in human and mouse atherosclerotic plaque, thus suggesting a mechanistic link between altered clock function and vascular pathologies [24,25]. Here, we will review our current knowledge on the relationship between cardiovascular diseases and the circadian clock. We will emphasize the impact of nuclear receptors (NRs) in this particular interaction. NRs are transcription factors that can be bound and activated by natural lipophilic ligands and by synthetic ligands, thus representing interesting both pharmacological targets to modulate the clock and common atherosclerosis risk factors.

## 2. The Molecular Clock and the Nuclear Receptor Superfamily

### 2.1. The Nuclear Receptor Superfamily

**Figure 2:**
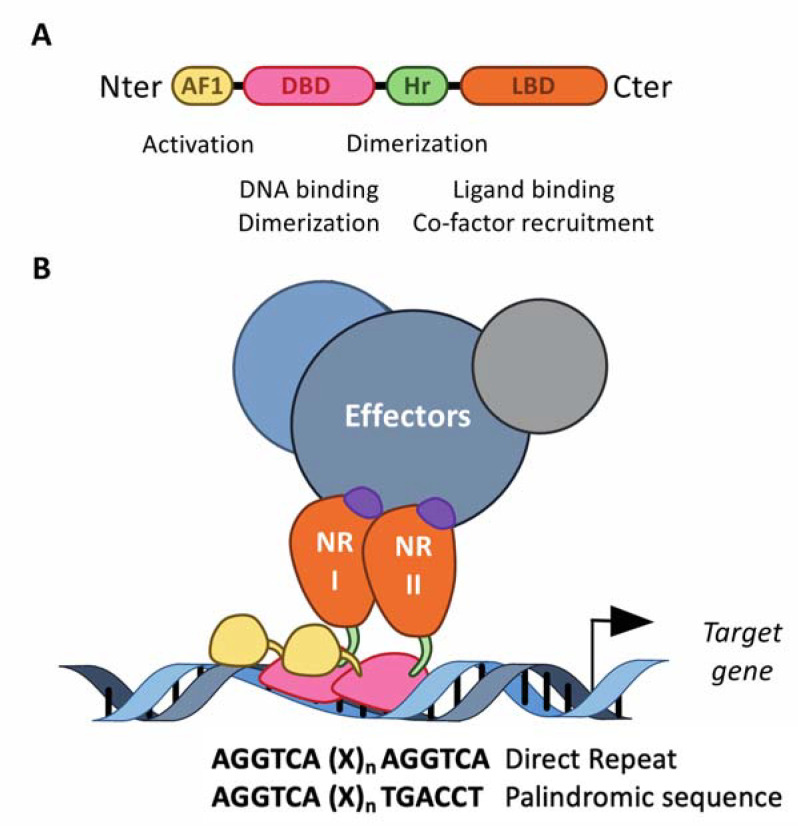
The structure of the nuclear receptors. (**A**) NRs consist of modular domains, including a variable amino N-terminal activation domain (AF-1), a highly conserved DNA-binding domain (DBD) and a conserved hinge region linking the DBD with the conserved ligand-binding domain (LBD). The DBD mediates the specific recruitment of NRs monomers, homodimers and heterodimers to their DNA response element and is involved in the dimerization of NRs with their partner, together with the hinge region and the LBD. (**B**) NRs usually work as homo- or heterodimers, which bind to a specific response element composed of two AGGTCA half-sites separated by one to four nucleotides in the promoter of target genes. These half-sites are organized either as a palindromic sequence or a direct repeat. The LBD mediates ligand-dependent interactions with effectors, namely transcriptional co-activators or co-repressors. These interactions are controlled by ligand-dependent conformational changes in the LBD. In absence of a ligand, co-repressors are preferentially bound to NRs, whereas ligand binding induces a conformational, which then triggers the release of co-repressors, allowing co-activators to bind.

The structurally conserved NRs class belongs to the transcription factor superfamily [26,27,28]. Interestingly, in addition to the DNA binding domain commonly present in the transcription factor structure, nuclear receptors also exhibit a ligand-binding domain (LBD) at their carboxy terminal extremity (Figure 2). NRs usually work as homo- or heterodimers, which bind to a specific response element composed of two AGGTCA half-sites separated by one to four nucleotides in the promoter of target genes (Figure 2). These consensus half-sites are organized either as a palindromic sequence or a direct repeat (Figure 2). The NR superfamily is sub-divided into four classes based on both their ligand- and DNA-binding properties and on the nature of their partner [29]. The heme receptors Rev-erb (Rev-erbα and Rev-erbβ) belong to the third NR class of adopted receptors. The NRs from this class act as monomers or homodimers bound on direct repeat response elements. Retinoic acid receptor-related orphan receptors (RORα, RORβ and RORγ) belong to the fourth class of orphan nuclear receptors. The LBD mediates ligand-dependent interactions with transcriptional co-activators, such as p300/CBP, or co-repressors, such as NCoR or SMRT. These interactions are controlled, at the structural level, by ligand-dependent conformational changes in the last α-helix 12 (αH12) of the LBD, known as AF2 [30]. In absence of a ligand, co-repressors are preferentially bound to NRs, especially those of the class II, including fatty-acid-activated peroxisome proliferator-activated receptors (PPARs), oxysterol-activated liver X receptors (LXR), 9-cis-retinoic acid- and all-trans-retinoic-acid-activated retinoic acid receptors (RAR), whereas ligand binding induces a conformational change in the αH12 helix, which then triggers the release of co-repressors, allowing co-activators to bind. If several NRs, especially those of the class II, are then able to bind target genes in absence of a ligand and recruit co-repressors to actively repress gene expression, class I steroid hormone receptors are usually sequestered into the cytoplasm in absence of a ligand, and translocate into the nucleus to bind their target genes in the presence of a ligand. Rev-erbα and Rev-erbβ lack the αH12 helix, which prevents the recruitment of co-activators [31,32]. Instead, Rev-erbs are able to recruit co-repressors and actively repress gene expression in absence of a ligand, and ligand binding enhances co-repressor recruitment and the transcriptional repressive activity of Rev-erbs to further inhibit the expression of their target genes [31,32]. Their transcriptional activity might be regulated by post-translational modifications, including phosphorylation, ubiquitination and SUMOylation [33,34,35,36,37,38,39,40,41,42].

### 2.2. The Biological Clock

Circadian rhythms are generated by a clock machinery present in most cell types, including endothelial cells, smooth muscle cells and immune cells [43,44,45,46,47]. The central pacemaker is located in the suprachiasmatic nucleus (SCN) of the hypothalamus, where it receives light information from the retina via the retino-hypothalamic tract [48]. The SCN pacemaker synchronizes peripheral clocks using humoral and neural outputs, notably through the hypothalamic–pituitary–adrenal axis. The release of the adrenocorticotropic hormone (ACTH) likely cooperates with the sympathetic and parasympathetic nervous system to regulate the oscillatory release of clock-resetting hormones (glucocorticoids, adrenaline and noradrenaline) from the adrenal gland. In addition to hormones, the sympathetic nervous system also directly innervates peripheral tissues and can directly modulate the activity of local clocks by controlling the rhythmic release of noradrenaline from nerve synapses [49]. Peripheral clocks are also synchronized by other time cues, such as food intake and exercise [49].

### 2.3. The Molecular Clock

**Figure 3:**
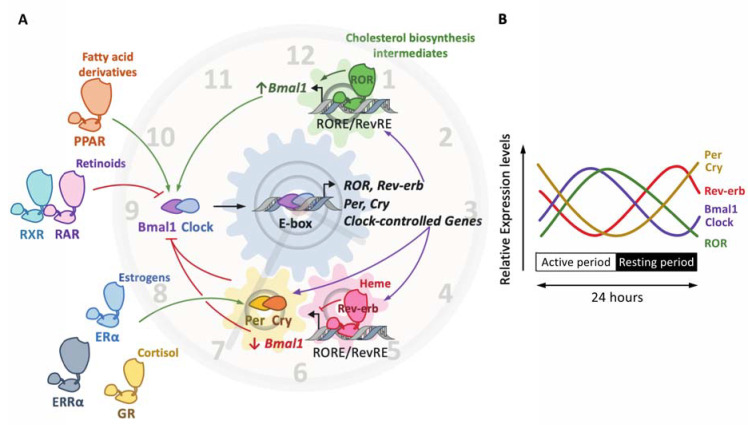
The molecular clock and the nuclear receptor superfamily. The molecular clockwork is composed of transcription–translation feedback loops. The transcription complex Bmal1/Clock induces the expression of E-box-containing genes, including the negative regulators period (Per) and cryptochrome (Cry) (**A**). In turn, the Per/Cry heterodimer inhibits the transcriptional activity of Bmal1/Clock. Once Per and Cry levels are sufficiently low, a new cycle may start. The Clock/Bmal1 heterodimer induces the expression of the nuclear receptors Rev-erbα/β and retinoic-acid-related orphan receptor α, β and γ (RORα/β/γ). Rev-erbs and RORs compete for the binding of RevRE/RORE elements in common target genes to repress or activate, respectively, their transcription. The overall effect of these loops is the rhythmic expression of these factors (as shown on (**B**)), thus generating a circadian expression pattern. Other nuclear receptors, which do not belong to the core clock, are, however, able to activate (green arrow) or inhibit (red arrow) the expression and/or the activity of core clock components. This includes the glucocorticoid receptor (GR) activated by cortisol, the peroxisome proliferator-activated receptors (PPARs) alpha and gamma, whose natural ligands are fatty acids and derivatives, the estrogen receptor (ER) alpha, whose natural ligands are estrogenic compounds, such as estradiol, estrone and estriol, the 9-cis-retinoic retinoid X receptor (RXR), the all-trans-retinoic receptor and 9-cis-retinoid retinoid activated receptor (RAR) and the estrogen-related receptor (ERR) alpha.

At the molecular level, the mammalian clock consists of a complex network of transcription factors and interconnected transcriptional feedback loops that generate rhythms with a period of around 24 h [50]. The positive limb is driven by the heterodimer Bmal1 (brain and muscle ARNT-like 1) and Clock (circadian locomotor output cycles kaput), which bind to E-boxes in the promoter of its target genes, including Per and Cry clock genes, in order to induce their transcription. Period (Per) 1/2/3 and Cryptochrome (Cry) 1/2 form the negative limb (Figure 3). Once they reach a sufficient threshold, Per and Cry heterodimerize and translocate to the nucleus, where they quench the Bmal1–Clock heterodimer in order to inhibit its transcriptional activity (Figure 3). In addition, the ligand-activated nuclear receptors Rev-erbs and RORs also finely tune this circuitry at the transcriptional level, as detailed below [46]. The clock components not only control each other’s transcription, but also modulate the expression of numerous genes, thereby generating rhythmic transcriptional oscillation in transcriptional programs and specific tissue functions, including in vascular cells.

The rhythmicity observed in gene transcription is also due to the circadian variation of epigenetic marks, namely histone modifications, and the subsequent chromatin organization at regulatory regions [50,51]. Besides, dynamic spatial and temporal chromatin architecture variation is an additional regulatory level of circadian genome function [52]. Finally, post-translational modifications, including phosphorylation [53], ubiquitination [54], SUMOylation [55], acetylation [56] and O-Glc-NAcylation, ensure diurnal fluctuations of the clock component stability and activity by controlling their degradation, localization and interaction with partners. Post-translational modifications thereby tune the pace and robustness of the clockwork [57].

### 2.4. Nuclear Receptors in the Heart of the Clockwork

#### 2.4.1. Nuclear Receptors as Core Clock Components

Rev-erbs act as transcriptional repressors whereas RORs are thought to compete with Rev-erbs for the same response element in order to induce the transcription of common target genes, including the Bmal1-encoding gene *Arntl* (Figure 3A) [58]. Importantly, Rev-erbs also repress *Cry1* transcription, thus controlling both limbs of the clock in a coordinated manner [59]. Intriguingly, because the ROR and Rev-erb NRs are not temporally co-expressed (Figure 3B), the yin and yang of their regulatory ballet may likely originate from a finely tuned regulation of the threshold level reached by each NR in order to supplant the other and to occupy their common response elements. Indeed, Rev-erb isotypes harbor a strong circadian rhythmicity in their abundance, leading to a rhythmic competition for the binding to the *Arntl* promoter that contributes to Bmal1 oscillations. Further investigations are needed to fully elucidate these underlying temporal mechanisms.

#### 2.4.2. Nuclear Hormone Receptors as Extrinsic Clock Modulators

In addition to Rev-erbs and RORs, other NRs, including the glucocorticoid receptor GR, are also able to control the clock [60,61] (Figure 3). The GR is activated by glucocorticoids, such as the human cortisone or the mouse corticosterone. Its production is located in the adrenal gland and is under the clock-dependent regulation of the adrenocorticotropic hormone, indicating that the glucocorticoid levels are a clock-regulated process [62]. Indeed, the circulating levels of the human cortisone or the mouse corticosterone display circadian rhythmicity, with peak levels during the onset of the activity period, indicating that the activation of the GR is circadian. Apart from its anti-inflammatory properties, a pivotal role of the GR in the clock machinery regulation has been uncovered in peripheral tissues. Indeed, the elevation of the glucocorticoid during the beginning of the activity period acts as a resetting cue for peripheral clocks and non-SCN hypothalamic nuclei [63]. At the molecular level, glucocorticoid response elements (GRE) have been identified in the promoter of several clock genes, including *Rev-erbα*, *Per1* and *Per2*, suggesting an important role of the GR in the regulation of the clock machinery [64,65] (Figure 3). In addition, Cry directly interacts with the GR and regulates its activity, as demonstrated by the increased response to dexamethasone, a GR ligand, in *Cry1*/*Cry2* double knockout mice [66]. Clock has also been shown to control GR activity by regulating GR acetylation, thus modulating GR recruitment to DNA [67]. In addition to the GR, the estrogen receptor ERα induces *Per2* gene expression in mammary glands and the uterus, also through a direct mechanism [68,69] (Figure 3). The class IV estrogen-related receptor ERRα is also involved in the control of the metabolic clock outputs by genetically interacting with Bmal1 in a PROX1-dependent manner [70] (Figure 3). Reciprocally, ERRα expression is affected by the clock machinery, highlighting the complex interplay between the ERRα and the clock machinery [71]. Interestingly, the expression of *Rev-erbα* is directly modulated by the binding of PPARα and PPARγ to a PPRE present in the promoter of *Rev-erbα* in the liver and in the adipose tissue, respectively [72,73] (Figure 3). In addition to *Rev-erbα*, PPARα also positively regulates the expression of *Arntl*/*Bmal1* in rodent liver [74], whereas PPARγ controls blood pressure and heart rate circadian pressure in the vasculature by regulating *Bmal1* expression as well [75]. Due to the fact that PPARα and PPARγ activity is controlled by fatty acids and derivatives [76], such mechanisms also reveal the intricate relationship between metabolic homeostasis and the circadian clock. In the same manner, all-trans retinoic acid was shown to reset the clock in the vasculature by inhibiting the Clock/Bmal1 complex activity in a RARα and retinoid X receptor (RXR)-α-dependent manner [77] (Figure 3).

## 3. Clock and Atherosclerosis

Many physiological processes, such as the management of energy intake or mobilization of energy storage, need to be gated at the most appropriate time window, and are then regulated in a circadian manner [46]. As mentioned above, a plethora of physiological processes are regulated in a circadian manner [46]. This includes pathways involved in the pathogenesis of atherosclerosis, such as endothelial cell function, hemostasis and lipid metabolism, as well as immune function, in a process known as circadian immunity [46]. Indeed, circadian rhythms not only influence systemic mediators, including immune cells and lipids, but also locally control cells within the vessel wall. For instance, gene profiling found that 5% to 10% of the transcriptome displays circadian expression patterns in mouse aortas [78]. In addition, several studies demonstrated the existence of a functional circadian clock in the vasculature [77,79,80].

### 3.1. Clock Disruption and Atherosclerosis: Clinical Evidences

Over the past hundred years of global industrialization, mankind underwent some important changes in its lifestyle, including its food habits, the ease of travel, the increase in shift work and social demands and erratic artificial light exposure from luminescent screens, which have dramatically altered circadian rhythms. In addition, elderly people display an altered circadian rhythm robustness, suggesting that aging may also be considered as an alteration factor of the circadian rhythm [81,82,83,84]. Disruption of the intrinsic molecular clock is now well recognized to have severe repercussions on health. Many pathogenic processes involved during atherogenesis are indeed worsened when the clock is altered (Figure 1). This will be discussed at the epidemiological, clinical and pre-clinical levels.

#### 3.1.1. Epidemiological Studies

Numerous clinical studies have reported that the alteration of circadian rhythms in humans represents an additional risk factor for metabolic and chronic inflammatory disorders, such as atherosclerosis [22,85]. For instance, the intimal-media thickness has been found to be increased in shift workers compared to day workers. Interestingly, this was associated with a 2.2-fold increase in the odds of carotid plaques, even after adjusting for age and risk factors, including socio-economic position, job strain, smoking, diet, family history, metabolic status and alcohol consumption [19] (Table 1). Strikingly, these observations were made in men aged under 40 years but not in women from the same age category. Similarly, social jet lag, i.e., a social behavior leading to differences between mid-sleep time on workdays and days off, may be associated with an increased cardiovascular risk, as indicated by a decreased high density lipoprotein cholesterol level, higher triglyceride levels and decreased insulin sensitivity [86,87] (Table 1). Social jet lag was also associated with a disturbed heart rate in men [88] (Table 1). Unexpectedly, however, shift work was not associated with any alteration in the heart rate [88]. The same observation was recently reported in a Japanese cohort, suggesting that shift work was not associated with an increased risk of atherosclerosis. Instead, lifestyle behavior, such as reduced physical exercise, was unsurprisingly associated with an increase in visceral fat, whereas habitual smoking was consistently associated with the presence of atherosclerosis in middle-aged male Japanese workers [89]. However, more recently, Peñalvo et al. compared the effect of different shift work patterns in the Aragon Worker’s Heath Study (AHWS) cohort of shift and day workers in middle-aged male subjects [90] (Table 1). They demonstrated that workers with the most intense rotating shift work (morning-evening-night) presented higher odds of subclinical atherosclerosis compared to other shift workers, independently of lifestyle and of the presence of metabolic risk factors [90]. Altogether, these studies emphasize the importance of the pattern of shift work on atherosclerosis, but they also highlight the difficulty in forming conclusions from the real-life cohort, as many parameters may influence the results, even after statistical adjustments. Such discrepancies highlighted here emphasize the difficulty to dissect the effects of circadian alteration and environmental factors, and this is particularly underlined by the variety and duration of shift work patterns of the different studies. In addition, although epidemiological studies have reported a morning peak in adverse cardiovascular events [15,16,91], such data were collected during regular sleep/wake cycles, which also prevent determining whether this is caused by the circadian system or by behavioral and environmental factors.

#### 3.1.2. Controlled Clinical Studies

In order to assess the role of the circadian machinery in vascular function and the cardiovascular risk, controlled acute and chronic circadian misalignment experiments were conducted in healthy human volunteers. In such protocols, volunteers are kept in stringently controlled light and energy intake conditions in order to create a constant routine or a forced desynchrony. Constant routine protocols usually consist of participants being kept awake under dim light, under semi-recumbent posture and with evenly spaced isocaloric meals, all for more than 24 h [93], whereas the forced desynchrony protocols are designed to uncouple the circadian machinery from the daily behavioral/environmental rhythms. As for the constant routine, the forced desynchrony allows us to then assess the circadian effect on physiological parameters, but also investigates the interaction of the clock with behaviors [93]. Forced desynchrony protocols usually consist of maintaining volunteers on a non-24 h sleep–wake cycle (either 20 h or 28 h) under dim light (below 5 lux to minimize the light effect on the biological clock). Using these controlled conditions, a circadian cycle has been observed in blood pressure, heart rate, platelet aggregability and immune response [93]. Interestingly, chronic and acute circadian misalignment obtained in forced desynchrony protocols increases cardiovascular disease risk factors [22,92]. Finally, it is noteworthy that circadian misalignment causes disrupted sleep, which is known to be associated with an increased risk of cardiovascular diseases, including atherosclerosis [94].

### 3.2. Contribution of Pre-Clinical Models in Our Understanding of the Clock Role in Atherosclerosis

From the clinical studies, it is then assumed that clock alteration promotes plaque development and atherosclerosis. Moreover, the rhythmic expression of clock genes is attenuated in human plaque-derived vascular smooth muscle cells [24], thus suggesting that pathological conditions, including atherosclerosis, alter the clock machinery in a reciprocal manner, making it more complicated to unravel the causal and the subsequent mechanisms involved in the crosstalk between the clock and the plaque. Pre-clinical studies using either environmental and genetic clock disruption models have been instrumental in this regard in identifying the underlying mechanisms.

#### 3.2.1. Contributions of Environmental Factors to Clock Alteration in Atherosclerosis

The jet-lag-induced disruption of the clock accelerates atherosclerosis and promotes the onset of a vulnerable plaque in *LDLr^−/−^* mice, a model prone to atherosclerosis [95] (Table 2). Interestingly, although the energetic balance, namely glucose and cholesterol levels, was not affected in mice with jet lag compared to controls, the molecular clock was disrupted in lesional macrophages, which was associated with an increase in ER stress and apoptosis [95]. In addition, the circadian disruption in pro-atherogenic *ApoE^−/−^* mice exposed to constant light exacerbates the lesion size in male mice, but not in female mice [96] (Table 2). Interestingly, this effect may be explained by an increase in circulating pro-atherogenic lipoproteins rather than by an impaired immune function [96]. As mentioned above, the estrogen receptor ERα is not only an important regulator of the clock machinery but also a critical atheroprotective factor [97,98,99,100,101,102], which may account for this gender-specific defense against circadian desynchronization-mediated atherogenesis. Furthermore, an increase in serum cholesterol levels was observed in *ApoE^−/−^* mice exposed to constant light, demonstrating here the impact of a severe clock disruption on cholesterol homeostasis [96]. Interestingly, such an observation was corroborated in a mild jet-lag-induced clock alteration model, where *ApoE^−/−^* mice were exposed to a 12-h phase shift every 2 weeks. These mice also exhibited larger lesions, together with an increase in plasma triglycerides and total cholesterol levels [103]. Contrasting results have however been reported, i.e., no effect of constant light on atherosclerosis development in APOE*3-Leiden.CETP mice [104] (Table 2). Nonetheless, the phase-shift (advance and delay) in the light—dark cycle promoted atherosclerosis development in this mouse model, the most deleterious impact having been obtained in weekly alternating light—dark cycles (12 h shifts) [105].

#### 3.2.2. Global Genetic Alteration of Clock Components in Atherosclerosis

The genetic alteration of the molecular clock contributes to increased pathological remodeling and vascular injury in *Bmal1^−/−^* and in the *Clock^Δ19/Δ19^* mutant [113] (Table 2). For instance, the transplantation of arteries from *Bmal1^−/−^* mice or from *Per1/2^−/−^* mice into wild-type mice triggers the development of atherosclerosis in the transplanted graft, emphasizing a critical role for peripheral circadian clocks in the development of atherosclerotic lesions [106]. Importantly, the disruption of the molecular clock impairs lipid homeostasis and results in inflammation, which both promote atherogenesis [114,115,116]. For instance, Bmal1 controls lipoprotein production and biliary cholesterol secretion. As expected, its deletion then leads to hyperlipidemia and atherosclerosis [107]. Accordingly, *ApoE^−/−^Clock^Δ19/Δ19^* mice exhibit an increased plasma cholesterol compared to controls, and larger atherosclerotic lesions, which are associated with an increased macrophage, smooth muscle cell (SMC) and collagen content [108] (Table 2). Interestingly, Clock was proposed to control cholesterol efflux in macrophages. Accordingly, *Clock* deficiency would impair the reverse cholesterol transport and lead to atherogenesis in *ApoE^−/−^* [108]. Furthermore, the global adenovirus-mediated overexpression of Cry1 in *ApoE^−/−^* mice protects against the development of atherosclerosis through a TLR/NF-κB-dependent mechanism [109]. Cry1 overexpression in *ApoE^−/−^* mice significantly reduces the expression of proinflammatory markers, and these mice display lowered total cholesterol, triglycerides and LDL cholesterol levels [109] (Table 2). Then, overexpressed Cry1 improves both inflammatory and lipid profiles, thus accounting for the global protection against atherogenesis. It is noteworthy that patients exhibiting atherosclerotic plaques display lower blood cell *Cry1* mRNA levels compared to the controls, acknowledging the protective effect of Cry1 against atherosclerosis in humans as well [109]. Accordingly, the deletion of *Cry1* and *Cry2* in mice leads to elevated pro-inflammatory cytokines and an increased iNOS [117]. This inflammatory effect may be due to the loss of interaction with the glucocorticoid receptor and the ensuing dysregulation of its downstream pathways [66]. In addition to pro-inflammatory effects, the deletion of both *Cry1* and *Cry2* promotes glucose intolerance and elevated plasma glucose levels in response to acute feeding after a 12 h overnight fasting in mice [66].

The immune system and the homeostasis of the cardiovascular system are intricately associated [47,114,115]. On the one hand, myeloid cells are recruited to atherosclerotic lesions in a circadian manner, with a peak during the active-to-rest transition, through the rhythmic deposit of CCL2 on the arterial endothelium by circulating cells (Figure 1). On the other hand, the circadian oscillations of this cellular recruitment are shifted by 12 h in healthy microvascular beds in order to reach a peak at the early active phase [118]. As such, a chrono-pharmacological approach targeting monocyte recruitment via the timed inhibition of the CCR2/CCL2 axis during the active phase dampened atherosclerotic lesion development. Since the immune function is regulated in a circadian manner, it is therefore not surprising that heart homeostasis and repair may be regulated in a circadian manner by the immune system. For instance, neutrophils are the first effector cells in innate immunity, and their blood counts oscillate in a circadian manner [119,120]. Interestingly, this feature is under the control of Bmal1 in neutrophils and has some consequences on vascular health if the renewal is impaired [121]. Indeed, neutrophils are also recruited to tissues following a daily rhythm under a steady state. In a mouse heart, for instance, a peak of migration is observed in the evening and is associated with a higher expression of chemokines, such as CCL2/MCP1, CXCL1 and CXCL5, and adhesion molecules, including ICAM1 and VCAM1 [122]. Interestingly, the proper recruitment of neutrophils to the tissues depends on the matching between the expression of these chemokine ligands in the tissue and the expression of the corresponding receptor CXCR2 in the evening, i.e., at the beginning of the active phase [122]. If the secretion and the production of both partners is mismatched, as in *Cxcr2* KO mice, for instance, or using a CXCR2 pharmacological inhibitor, then the recruitment is impaired [122]. Importantly, the induction of an experimental myocardial infarct at the peak of cardiac neutrophil recruitment, in the evening, results in a significantly higher infarct size and compromises the cardiac healing, as well as the heart function [122]. Intriguingly, monocytes also exhibit a circadian recruitment pattern to the mouse heart and display an elevated expression of the CCR2 receptor in the evening as well, which promotes a higher cardiac infiltration relative to the day [123]. In addition to Bmal1, Clock is also involved in this process. Indeed, the infarct size, as well as the infiltration of neutrophils and monocytes, are increased in mice expressing the *Clock^Δ19/Δ19^* mutant [124]. Intriguingly, as the gut physiology affects the circadian system, Mistry et al. have demonstrated that an intact gut physiology is important for cardiac repair after a myocardial infarction [125]. Indeed, a disrupted microbiome impairs the inflammatory response in infarcted myocardium and heart repair [125]. Strikingly, this effect was controlled by Clock, as shown in *Clock^Δ19/Δ19^* mice [125]. In *ApoE^−/−^* mice, the adhesion and infiltration of neutrophils and monocytes into atherosclerotic plaques also depends on CCR2 expression, and displays a diurnal rhythm as well; however, it peaks at the end of the active phase and the beginning of the resting phase, i.e., in the morning in mice [118,126]. In this context, myeloid cells, namely monocytes and neutrophils, deposit CCL2 on large arteries in a cyclic manner, which would then account for the cyclic recruitment of myeloid cells to atherosclerotic plaque [118]. Importantly, Winter et al. demonstrated that this was due to the mere expression of Bmal1 in the myeloid compartment, but not in endothelial cells, thus suggesting that the immune cells, in this context, generate their own tools in order to cyclically infiltrate the vascular wall [118].

#### 3.2.3. Cell-Specific Clock Gene Alteration in Atherosclerosis and Cardiovascular Diseases

Studies in experimental clock alteration or in whole-body knock-out mice highlight an overall role of the clock during atherogenesis. The use of tissue-specific deficient mouse models for clock components, however, brings further insights into the specific function of the clock in vascular cells and pro-atherogenic partners.

For instance, *Bmal1^−/−^*, as well as *Per2^−/−^* mice, display impaired acetylcholine-induced vasorelaxation, probably due to an impaired Akt/eNOS signaling pathway, which is suggestive of an important endothelial-specific regulatory function of the vascular tone [113,127]. Interestingly, the endothelial-specific deletion of *Bmal1^−/−^* alters blood pressure, heart rate and heart activity, but not their diurnal variations in mice [128] (Table 3). Furthermore, Clock overexpression directly enhances the expression of adhesion molecules, such as VCAM1 and ICAM1, in mouse microvascular cells, whereas clock ablation inhibits ICAM1 expression, thereby regulating monocyte adhesion to endothelial cells [129]. Finally, Per2 is also associated with aortic endothelial dysfunction, as shown after its ablation in mice, which exhibited a decreased nitric oxide and vasodilatory prostaglandin production, as well as an increased release of the cyclooxygenase 1-derived vasoconstrictor [127].

In humans, as mention above, the circadian expression pattern of core clock genes *Bmal1*, *Clock*, *Per*, *Cry* and *Rev-erbα* is dramatically attenuated in atherosclerotic plaques in both men and women [24]. Interestingly, healthy SMC were isolated from younger donors compared to SMC isolated from human plaque, which could have been a confounding factor [83]. However, even when adjusting for age, the amplitude of rhythmic oscillations in the clock components is still dampened in plaque-derived SMC compared to healthy ones [24]. Additionally, smooth muscle-specific *Bmal1^−/−^* mice display a reduced amplitude in blood pressure oscillations along the day when compared to control mice, and are protected from abdominal aortic aneurysm [130,131] (Table 3). The effect of the SMC-specific ablation of core clock genes on atherogenesis still needs to be investigated.

In the immune system, the myeloid deletion of *Bmal1* enhances the recruitment of Ly6C^hi^ monocytes and then worsens atherosclerosis [132] (Table 3). However, intriguingly, it has been reported lately that *Bmal1* ablation in the myeloid compartment delays plaque formation in proatherogenic *LDLr^−/−^* mice and is associated with a decrease in pro-inflammatory pathways and an increased recruitment of anti-inflammatory CD206+ macrophages that are known to stabilize lesions [133] (Table 3). Such a discrepancy may originate from the different strains used as the control. Indeed, the latter used *LysM^Cre/+^Ldlr^−/−^* as control [133], whereas the former compared *ApoE^−/−^Bmal1^Fl/Fl^LysM^Cre/+^* with *ApoE^−/−^Bmal1^Fl/Fl^* [132]. As the *LysM^Cre/+^* heterozygous mice may have their own phenotype in a dyslipidaemic background, additional experiments are warranted to definitively conclude the myeloid-specific role of *Bmal1* in atherosclerosis. Furthermore, *Cry1/Cry2* deficiency in the bone marrow protects from atherogenesis in *LDLr^−/−^* mice [134] (Table 3). Interestingly, cryptochrome deletion alters macrophage function in vitro, namely phagocytosis and efferocytosis, MCP-1 secretion and LDL uptake, thus lowering foam cell formation [134]. In addition, while oxidized LDLs (oxLDL) decrease the expression of core clock genes, such as Bmal1 and Rev-erbα, in synchronized primary macrophages, this effect was abolished in Cry1/2 knock-out macrophages, demonstrating a key effect of cryptochromes in the response of macrophages to oxLDL stimuli [134]. This might be due to the control of the LDL receptor by Cry. Indeed, the circadian rhythmicity of *LDLr* is abolished in Cry1/2-deficient macrophages, which may explain the lack of effect of oxLDL in macrophages [134]. Strikingly, the rescue of *LDLr* mRNA oscillations was shown to enhance modified LDL internalization compared to a non-oscillatory *LDLr* mRNA in macrophages, thus demonstrating the importance of a proper circadian clockwork to optimize cellular function [134]. In this study, Cry1/2 then appear to play deleterious effects on atherogenesis when they are both altered in myeloid cells. Interestingly, previous studies demonstrated a global beneficial effect of Cry1/2 in global knock-out mice, thus emphasizing the relevance of such comparative studies and the importance in targeting the right cells/tissues in future therapeutical strategies in order to avoid adverse effects.

### 3.3. Post-Transcriptional Control of the Clock in Atherogenesis

Apart from a direct control of gene transcription by the clock, post-transcriptional pathways have been involved in the impact of the clock on atherogenesis. For instance, the upregulation of *miR155* increases the atherosclerosis lesion size, cell apoptosis, total triglyceride and cholesterol levels by inhibiting *Bmal1* expression in *ApoE^−/−^* mice. Importantly, *miR155* impairs aorta diastolic function, thus suggesting an important regulatory function in endothelial cells and vasoconstriction [136]. Interestingly, the microRNA *miR21* expression displays diurnal oscillations and was suggested to be regulated by the molecular clock. Strikingly, *miR21* imposes a diurnal rhythm of apoptosis in an XAF1-manner in atherosclerotic lesions. Interestingly, such a deregulation would lead to the desynchronization between apoptosis and efferocytosis, which would then lead to an increase in the necrotic core size [137]. It may then be assumed that the clock machinery could control the vascular wall fate by indirectly regulating the expression of atherogenic key factors as well through miRNA-dependent mechanisms. Such events are far to be fully described and need further investigations.

## 4. Core Clock Components Rev-Erb and ROR in Atherosclerosis

Many NRs have been involved in the development of cardiovascular diseases and atherosclerosis [76,138,139]. Due to being able to be activated by natural, as well as synthetic, molecules, NRs are attractive therapeutic targets to directly modulate the expression of genes/pathways of interest. However, since Rev-erbs and RORs are the only two NR subfamilies belonging to the core clock components [47,140], we will focus on these NRs.

The impact of Rev-erbs and RORs has already been reported in mouse models of atherosclerosis. For instance, the shRNA-mediated deletion of Rev-erbα, specifically in hematopoietic cells, exacerbates the development of atherosclerotic lesions in *LDLr^−/−^* mice [135] (Table 3). At the cellular level, the ablation of Rev-erbα in bone marrow-derived monocytes promotes the skewing of pro-inflammatory macrophages toward an anti-inflammatory phenotype [135]. Interestingly, the knock-down of *REV-ERBα* exacerbates a pro-inflammatory phenotype in LPS-primed human monocyte-derived macrophages [141]. Accordingly, the treatment of mice with the synthetic Rev-erb agonist SR9009 reduces the atherosclerotic lesion size and triggers the expression of macrophage anti-inflammatory markers [111] (Table 2). In addition, treatment of mouse primary macrophages with the Rev-erb natural ligand heme [142] induces an alternative differentiation into an anti-inflammatory phenotype [135]. This suggests that targeting Rev-erb using pharmacological compounds would represent an attractive strategy against atherosclerosis. However, because the SR9009 compound displays Rev-erb-independent effects [143,144], additional compounds, such as SR10067 or GSK1362, have been developed [145,146], and should be tested in this setting. The homozygous staggerer (*sg/sg*) mutant mouse harboring a deletion in the *RORα* gene displays neurodegeneration and exhibits exaggerated immune activity, such as inflammatory cytokine hyperproduction. Strikingly, *sg/sg* mice that are fed a western diet, but without being bred with pro-atherogenic mice, such as *ApoE^−/−^*or *LDLr^−/−^*, develop severe atherosclerotic lesions, which is associated with a dramatic decrease in intestinal *ApoA-I* and *ApoA-II* mRNA, lower levels of circulating ApoA-I and ApoA-II and a substantial alteration of HDL cholesterol levels [110] (Table 2). Finally, the inhibition of RORα and RORγ using the inverse agonist SR1001 suppresses atherosclerosis in *LDLr^−/−^* that are fed a high cholesterol diet [112] (Table 2). Unexpectedly, ROR inhibition affects LDL, but not HDL levels [112]. In addition, ROR inhibition reduces Th17 cell levels and increases the quantity of Treg and Th2 cells, thereby enhancing an anti-atherogenic immune profile [112].

Both RORs and Rev-erbs play an important regulatory role in the control of metabolic homeostasis (see for review [147,148,149]. Importantly, Rev-erbα is involved in the recycling of cholesterol by promoting biliary acid synthesis [150]. Such a feature could then indirectly link Rev-erbα to the reverse cholesterol transport and help to reduce macrophage cholesterol levels. Interestingly, RORα directly enhances the expression of the apolipoprotein A1, the main component of the high-density lipoprotein, thereby suggesting a role of RORα in the reverse cholesterol transport as well [151]. It is noteworthy that in mice and humans, Rev-erbα controls the expression of the pro-atherogenic apolipoprotein ApoC-III [152,153], which is currently considered to be a very potent therapeutic target to treat adverse cardiovascular events, and is the main focus of an extensive current research [154]. Moreover, RORα appears to enhance the expression of *ApoC-III*, as shown in *sg/sg* mice, which exhibit lower *ApoC-III* mRNA and circulating protein levels [155]. As shown for Rev-erbα, RORα is directly recruited to the *ApoC-III* promoter in order to activate *ApoC-III* transcription [155].

In addition, Rev-erbα controls the circadian expression of cytokines, including IL6, IL-1β and CCL2/MCP1, which are key pro-atherogenic factors [156,157]. Furthermore, RORα inhibits the expression of pro-inflammatory genes, including IL-6, IL-8 and COX-2, in human primary SMCs. At the molecular level, RORα negatively interferes with the NF-κB signaling by inducing the expression IκBα [158].

At the molecular level, Rev-erbα and RORγ have been shown to control the expression and activity of the NLRP3 inflammasome [157,159]. The NLRP3 inflammasome is a master regulator belonging to the innate immune system, especially in macrophages, and a key sensor involved in maintaining cellular health in response to cytolytic pathogens or stress signals. The NLRP3 inflammasome is a cytoplasmic complex that is typically composed of a sensor molecule, such as NOD-like receptors (NLRs), an adaptor protein, including ASC, and an effector protein, such as caspase 1. Upon stimulation, inflammasome complex components associate to promote both the cleavage of the pro-caspase 1 into active caspase-1 and the subsequent activation of pro-inflammatory cytokines, including IL-18 and IL-1β. The deficiency or overactivation of such an important sensor leads to many diseases, including atherosclerosis. Indeed, the ablation or inhibition of the NLRP3 inflammasome pathway decreases atherosclerosis progression, thus emphasizing the critical role of NLRP3 in atherosclerosis initiation [160,161]. In fact, oxLDLs are internalized and promote both priming and the cholesterol crystals-mediated activation of NLRP3 in a CD36-dependent manner [162,163]. Inflammasomes are tightly controlled by a two-step activation regulatory process consisting of a priming step, which activates the transcription of inflammasome components, and an activation step, which leads to the inflammasome complex formation and the subsequent cleavage of pro-IL1 cytokines. Typical activators of the NLRP3 inflammasome are ATP-derived necrotic cells, ion fluxes, cathepsin released after crystal-induced lysosomal damage and mitochondrial ROS formation [140]. Apart from the NF-κB pathway, NRs have recently been proposed as additional regulators of this pathway [140].

The control of the NLRP3 inflammasome pathway by the clock was firstly reported in vivo and in vitro in both mouse and human primary macrophages [157]. In this context, NLRP3, IL-1β and IL-18 mRNA exhibit circadian oscillations in mouse peritoneal macrophages and in synchronized primary mouse and human macrophages, with a functional consequence on the secretion of these cytokines, which display a rhythmic pattern as well [157]. At the molecular level, oscillations in the NLRP3 inflammasome-related genes depend on the direct binding of both Rev-erbα [157] and RORγ [159] in macrophages. Interestingly, these two nuclear receptors are both recruited to the same site in the promoter of *Nlrp3*, thereby demonstrating that Rev-erbα and RORγ directly control the priming of NLRP3. In addition, Rev-erbα has been suggested to indirectly regulate the inflammasome priming by interacting with the NF-κB signaling pathway [164,165]. Furthermore, Rev-erbα would also prevent the NLRP3 activation step. Indeed, nigericin- and ATP-induced ASC speck formation was increased in *Rev-erbα-*deficient mouse primary macrophages compared to the control [157]. However, as this effect may only reflect the increase in *Nlrp3* gene expression, additional experiments are needed to uncover the underlying mechanisms. However, because Rev-erbα regulates mitochondrial function and autophagy processes [166], it may be speculated that Rev-erbα-regulated NLRP3 assembly is mediated by a decrease in ROS production and an improvement of the mitochondrial function. Finally, Rev-erbα is also involved in the control of the circadian expression of the long non-coding RNA *Platr4*, which serves as a circadian repressor of the NLRP3 inflammasome as well [167]. However, because *Platr4* and *Nlrp3* expression displays the same circadian expression pattern, further studies are needed to investigate the kinetics of *Platr4*-mediated *Nlrp3* mRNA stability. It is noteworthy that Rev-erb activation by natural or synthetic ligands reduces the secretion of IL-1β and IL-18 by inhibiting the expression of NLRP3 inflammasome component-related genes [157].

Interestingly, the NF-κB-driven long non-coding RNA Lnc-UC has lately been shown to be induced by the core clock component Bmal1, thereby generating the circadian expression of Lnc-UC [168]. Lnc-UC physically interacts with the Cbx1 protein to reduce its gene silencing activity via H3K9me3, thereby enhancing Rev-erbα expression in an epigenetic manner [168]. Then, by inducing Rev-erbα expression, Lnc-UC ablates NF-κB signaling and NLRP3 inflammasome signaling in macrophages [168].

## 5. Conclusions

Most pathogenic mechanisms, including cardiovascular and inflammatory diseases, are intricately associated with clock alteration. As a consequence, drug delivery at a specific time of day, i.e., chronotherapy, may prove beneficial to treat these diseases by enhancing drug efficiency and reducing adverse effects [169]. This is already widely used in cancer therapy, but is still struggling to impose itself in the field of cardiology and cardiovascular diseases. Besides, directly targeting clock components represents an attractive idea. Intuitively, it then appears that the optimal timing of drug administration coincides with the maximum peak of the target expression. It should be mentioned, however, that pathological tissues may exhibit shifted circadian rhythms compared to the healthy region, offering novel opportunities to dampen the damaging pathway at specific times of day in specific tissues/territories.

## Figures and Tables

**Table 1:** Clock alteration on atherogenesis in epidemiological and controlled clinical studies.

Clock Alteration Conditions	Group Specificities	Effect on Atherogenic Parameters	References
***Epidemiological Studies***			
Shift workers	Male, over 40-year-old, no women	Increased intimal-media thickness	[19]
Social jet lag	Male	Decreased HDL and insulin sensitivity, increased triglyceride and disturbed heart rate	[86,87,88]
Shift workers	Mid-aged male	Intense work shift induces higher odds of subclinical atherosclerosis	[90]
***Controlled Clinical Studies***			
Forced desynchrony	Acute and chronic circadian misalignment	Increased cardiovascular risk factors, including pro-inflammatory markers	[22,92]

**Table 2:** Pre-clinical models of global clock alteration.

Global Pre-Clinical Models					
Clock Alteration Models	Atherogenic Models	Cell-Specific Models	Effect on Atherogenesis	Molecular and Cellular Mechanisms	References
***Environmental Models***					
*Jet lag*	*LDLr^-/-^*	n/a	Induction	Alteration of clock in lesional macrophages, but no effect on energy homeostasis.	[95]
Mild jet lag	*ApoE^-/-^*	n/a	Induction	Increase in plasma TG and cholesterol.	[103]
Constant light	*ApoE^-/-^*	n/a	Induction	Increased lipoprotein circulation and serum cholesterol level, but no effect on immune system. Gender specificity (male).	[96]
Constant light	ApoE*3-Leiden	n/a	No effect	n/a	[104]
Phase shift in LD cycle	ApoE*3-Leiden	n/a	Induction	n/a	[105]
***Genetic Models***					
Common carotid artery transplant from *Bmal1^−/−^* or *Per1^−/−^* to WT mice	WT	Transplant of artery portion	Induction	Increased recruitment of macrophages and lymphocytes into the neointima.	[106]
*Bmal1^−/−^*	*ApoE^−/−^* and *LDLr^−/−^*	Global knock-out	Induction	Increased ApoB-containing lipoprotein circulation by reducing the SHP transcription factor expression, which then induces the Mtp expression. Decreased cholesterol excretion by inhibiting the GATA4 transcription factor expression and thereby Abcg5/Abcg8 expression.	[107]
*Bmal1* over-expression	*ApoE^−/−^* and *LDLr^−/−^*	Adenovirus-mediated liver-specific overexpression	Reduction	n/a	[107]
*Clock^Δ19/Δ19^*	*ApoE^−/−^*	Global impaired Clock protein function	Induction	Increased plasma cholesterol and increased macrophage, SMC and collagen content in atherosclerotic lesions. Impaired reverse cholesterol transport.	[108]
*Cry1* over-expression	*ApoE^−/−^*	Global adenovirus-mediated overexpression	Reduction	Reduced pro-inflammatory markers. Lowered total cholesterol, triglycerides and LDL cholesterol. TLR/NF-κB-dependent mechanism.	[109]
*sg/sg* mutant mouse	None	Global impaired RORα protein function	Induction	Increased intestinal *ApoA-I* and *ApoA-II* expression and reduced HDL cholesterol.	[110]
Rev-erb activation	*LDLr^−/−^*	SR9009 ligand	Reduction	Skewed from pro-inflammatory to anti-inflammatory macrophages.	[111]
ROR inhibition	*LDLr^−/−^*	SR1001 inverse agonist	Reduction	Decreased LDL levels and Th17 cell levels, and increased Treg and Th2 cell levels.	[112]

**Table 3:** Pre-clinical models of cell-specific clock alteration.

Cell-Specific Pre-Clinical Models					
Clock Alteration Models	Atherogenic Models	Cell-Specific Models	Effect on Atherogenesis	Molecular and Cellular Mechanisms	References
*Bmal1^EC^* *^−/−^*	n/a	Endothelial cell and Haematopoietic cells Tek^Cre^ mouse model	n/a	Alteration of blood pressure and heart rate/activity.	[128]
*Bmal1^SMC^* *^−/−^*	n/a	Smooth muscle cells αSM22^Cre^ mouse model	n/a	Reduced amplitude in blood pressure oscillation. Protects from abdominal aortic aneurism.	[130,131]
*Bmal1^Myeloid^* *^−/−^*	*ApoE^-/-^*	Myeloid cells LysM^Cre^ mouse model	Induction	Enhanced Ly6C^High^ monocyte recruitment.	[132]
*Bmal1^Myeloid^* *^−/−^*	*LDLr* *^−/−^*	Myeloid cells LysM^Cre^ mouse model	Reduction	Decreased pro-inflammatory pathways. Increased recruitment of anti-inflammatory CD206+ macrophages.	[133]
*Bmal1* *^−/−^*	*ApoE^−/−^* and *LDLr^-/-^*	Liver-specific knock-out	Induction	Increased ApoB-containing lipoprotein circulation by reducing the SHP transcription factor expression, which then induces the Mtp expression. Decreased cholesterol excretion by inhibiting the GATA4 transcription factor expression and thereby Abcg5/Abcg8 expression.	[107]
*Cry1,Cry2^BM^* *^−/−^*	*LDLr* *^−/−^*	Bone marrow transplant	Reduction	Alteration of macrophage functions, including phagocytosis, efferocytosis, MCP-1 secretion and LDL uptake.	[134]
*Rev-erbα^BM^* *^−/−^*	*LDLr* *^−/−^*	Transplant of shRev-erbα-infected bone marrow	Induction	Skewing from pro-inflammatory to anti-inflammatory macrophages.	[135]

## References

[1] Libby P., Buring J.E., Badimon L., Hansson G.K., Deanfield J., Bittencourt M.S., Tokgözoğlu L., Lewis E.F. (2019). Atherosclerosis. Nat. Rev. Dis. Primers.

[2] Galkina E., Ley K. (2009). Immune and Inflammatory Mechanisms of Atherosclerosis. Annu. Rev. Immunol..

[3] Hansson G.K., Hermansson A. (2011). The immune system in atherosclerosis. Nat. Immunol..

[4] Franck G., Even G., Gautier A., Salinas M., Loste A., Procopio E., Gaston A.-T., Morvan M., Dupont S., Deschildre C. (2018). Haemodynamic stress-induced breaches of the arterial intima trigger inflammation and drive atherogenesis. Eur. Heart J..

[5] Souilhol C., Serbanovic-Canic J., Fragiadaki M., Chico T.J., Ridger V., Roddie H., Evans P.C. (2020). Endothelial responses to shear stress in ather-osclerosis: A novel role for developmental genes. Nat. Rev. Cardiol..

[6] Xu S., Ilyas I., Little P.J., Li H., Kamato D., Zheng X., Luo S., Li Z., Liu P., Han J. (2021). Endothelial Dysfunction in Atherosclerotic Cardiovascular Diseases and Beyond: From Mechanism to Pharmacotherapies. Pharmacol. Rev..

[7] Tabas I., García-Cardeña G., Owens G.K. (2015). Recent insights into the cellular biology of atherosclerosis. J. Cell Biol..

[8] Chatzizisis Y.S., Coskun A.U., Jonas M., Edelman E., Feldman C.L., Stone P.H. (2007). Role of Endothelial Shear Stress in the Natural History of Coronary Atherosclerosis and Vascular Remodeling: Molecular, Cellular, and Vascular Behavior. J. Am. Coll. Cardiol..

[9] Moore K.J., Tabas I. (2011). Macrophages in the Pathogenesis of Atherosclerosis. Cell.

[10] Quillard T., Franck G., Mawson T., Folco E., Libby P. (2017). Mechanisms of erosion of atherosclerotic plaques. Curr. Opin. Lipidol..

[11] Sedding D.G., Boyle E.C., Demandt J.A.F., Sluimer J., Dutzmann J., Haverich A., Bauersachs J. (2018). Vasa Vasorum Angiogenesis: Key Player in the Initiation and Progression of Atherosclerosis and Potential Target for the Treatment of Cardiovascular Disease. Front. Immunol..

[12] Canet-Soulas E., Bessueille L., Mechtouff L., Magne D. (2021). The Elusive Origin of Atherosclerotic Plaque Calcification. Front. Cell Dev. Biol..

[13] Millar-Craig M.W., Bishop C.N., Raftery E.B. (1978). Circadian variation of blood-pressure. Lancet.

[14] Tofler G.H., Brezinski D., Schafer A.I., Czeisler C.A., Rutherford J.D., Willich S.N., Gleason R.E., Williams G.H., Muller J.E. (1987). Concurrent Morning Increase in Platelet Aggregability and the Risk of Myocardial Infarction and Sudden Cardiac Death. N. Engl. J. Med..

[15] Muller J.E., Stone P.H., Turi Z.G., Rutherford J.D., Czeisler C.A., Parker C., Poole W.K., Passamani E., Roberts R., Robertson T. (1985). Circadian Variation in the Frequency of Onset of Acute Myocardial Infarction. N. Engl. J. Med..

[16] Muller E.J., Ludmer P.L., Willich S.N., Tofler G.H., Aylmer G., Klangos I., Stone P.H. (1987). Circadian variation in the frequency of sudden cardiac death. Circulation.

[17] Suárez-Barrientos A., López-Romero P., Vivas D., Castro-Ferreira F., Núñez-Gil I., Franco E., Ruiz-Mateos B., Garcia-Rubira J.C., Fernandez-Ortiz A., Macaya C. (2011). Circadian variations of infarct size in acute myocardial infarction. Heart.

[18] Geiger S.S., Curtis A.M., O’Neill L.A.J., Siegel R.M. (2019). Daily variation in macrophage phagocytosis is clock-independent and dispensable for cytokine production. Immunology.

[19] Puttonen S., Kivimäki M., Elovainio M., Pulkki-Råback L., Hintsanen M., Vahtera J., Telama R., Juonala M., Viikari J.S., Raitakari O.T. (2009). Shift work in young adults and carotid artery intima–media thickness: The Cardiovascular Risk in Young Finns study. Atherosclerosis.

[20] Skogstad M., Mamen A., Lunde L.-K., Ulvestad B., Matre D., Aass H.C.D., Øvstebø R., Nielsen P., Samuelsen K.N., Skare Ø. (2019). Shift Work Including Night Work and Long Working Hours in Industrial Plants Increases the Risk of Atherosclerosis. Int. J. Environ. Res. Public Health.

[21] Jankowiak S., Backé E., Liebers F., Schulz A., Hegewald J., Garthus-Niegel S., Nübling M., Blankenberg S., Pfeiffer N., Lackner K.J. (2016). Current and cumulative night shift work and subclinical atherosclerosis: Results of the Gutenberg Health Study. Int. Arch. Occup. Environ. Health.

[22] Morris C.J., Purvis T.E., Hu K., Scheer F.A.J.L. (2016). Circadian misalignment increases cardiovascular disease risk factors in humans. Proc. Natl. Acad. Sci. USA.

[23] Dominguez F., Fuster V., Fernández-Alvira J.M., Friera L.F., López-Melgar B., Blanco-Rojo R., Fernández-Ortiz A., García-Pavía P., Sanz J., Mendiguren J.M. (2019). Association of Sleep Duration and Quality With Subclinical Atherosclerosis. J. Am. Coll. Cardiol..

[24] Lin C., Tang X., Zhu Z., Liao X., Zhao R., Fu W., Chen B., Jiang J., Qian R., Guo D. (2014). The rhythmic expression of clock genes attenuated in human plaque-derived vascular smooth muscle cells. Lipids Health Dis..

[25] Xu C., Lu C., Hua L., Jin H., Yin L., Chen S., Qian R. (2009). Rhythm changes of clock genes, apoptosis-related genes and atherosclerosis-related genes in apolipoprotein E knockout mice. Can. J. Cardiol..

[26] Green S., Walter P., Kumar V., Krust A., Bornert J.-M., Argos P., Chambon P. (1986). Human oestrogen receptor cDNA: Sequence, expression and homology to v-erb-A. Nat. Cell Biol..

[27] Hollenberg S.M., Weinberger C., Ong E.S., Cerelli G., Oro A., Lebo R., Thompson E.B., Rosenfeld M.G., Evans R. (1985). Primary structure and expression of a functional human glucocorticoid receptor cDNA. Nat. Cell Biol..

[28] Evans R.M., Mangelsdorf D.J. (2014). Nuclear Receptors, RXR, and the Big Bang. Cell.

[29] Mangelsdorf D.J., Thummel C., Beato M., Herrlich P., Schütz G., Umesono K., Blumberg B., Kastner P., Mark M., Chambon P. (1995). The nuclear receptor superfamily: The second decade. Cell.

[30] Rosenfeld M.G., Lunyak V.V., Glass C.K. (2006). Sensors and signals: A coactivator/corepressor/epigenetic code for integrating sig-nal-dependent programs of transcriptional response. Genes Dev..

[31] Feng D., Liu T., Sun Z., Bugge A., Mullican S.E., Alenghat T., Liu X.S., Lazar M.A. (2011). A Circadian Rhythm Orchestrated by Histone Deacetylase 3 Controls Hepatic Lipid Metabolism. Science.

[32] Yin L., Lazar M.A. (2005). The Orphan Nuclear Receptor Rev-erbα Recruits the N-CoR/Histone Deacetylase 3 Corepressor to Regulate the Circadian Bmal1 Gene. Mol. Endocrinol..

[33] Pourcet B., Torra I.P., Derudas B., Staels B., Glineur C. (2010). SUMOylation of Human Peroxisome Proliferator-activated Receptor α Inhibits Its Trans-activity through the Recruitment of the Nuclear Corepressor NCoR. J. Biol. Chem..

[34] Ghisletti S., Huang W., Ogawa S., Pascual G., Lin M.E., Willson T.M., Rosenfeld M.G., Glass C.K. (2007). Parallel SUMOylation-dependent pathways mediate gene- and signal-specific transrepression by LXRs and PPARgamma. Mol. Cell.

[35] Pascual G., Fong A.L., Ogawa S., Gamliel A., Li A.C., Perissi V., Rose D.W., Willson T.M., Rosenfeld M.G., Glass C.K. (2005). A SUMOylation-dependent pathway mediates transrepression of inflammatory response genes by PPAR-gamma. Nature.

[36] Saijo K., Winner B., Carson C.T., Collier J.G., Boyer L., Rosenfeld M.G., Gage F.H., Glass C.K. (2009). A Nurr1/CoREST Pathway in Microglia and Astrocytes Protects Dopaminergic Neurons from Inflammation-Induced Death. Cell.

[37] Blanquart C., Mansouri R., Fruchart J.-C., Staels B., Glineur C. (2004). Different ways to regulate the PPARalpha stability. Biochem. Biophys. Res. Commun..

[38] Gage M.C., Bécares N., Louie R., Waddington K.E., Zhang Y., Tittanegro T.H., Rodríguez-Lorenzo S., Jathanna A., Pourcet B., Pello O.M. (2018). Disrupting LXRα phosphorylation promotes FoxM1 expression and modulates atherosclerosis by inducing macrophage proliferation. Proc. Natl. Acad. Sci. USA.

[39] Becares N., Gage M.C., Voisin M., Shrestha E., Martin-Gutierrez L., Liang N., Louie R., Pourcet B., Pello O.M., Luong T.V. (2019). Impaired LXRα Phosphorylation Attenuates Progression of Fatty Liver Disease. Cell Rep..

[40] Torra I.P., Ismaili N., Feig J.E., Xu C.-F., Cavasotto C., Pancratov R., Rogatsky I., Neubert T.A., Fisher E.A., Garabedian M.J. (2008). Phosphorylation of Liver X Receptor α Selectively Regulates Target Gene Expression in Macrophages. Mol. Cell. Biol..

[41] Blanquart C., Barbier O., Fruchart J.C., Staels B., Glineur C. (2002). Peroxisome proliferator-activated receptor alpha (PPARalpha) turnover by the ubiquitin-proteasome system controls the ligand-induced expression level of its target genes. J. Biol. Chem..

[42] Berrabah W., Aumercier P., Lefebvre P., Staels B. (2011). Control of nuclear receptor activities in metabolism by post-translational modi-fications. FEBS Lett..

[43] Curtis A.M., Bellet M.M., Sassone-Corsi P., O’Neill L.A. (2014). Circadian Clock Proteins and Immunity. Immunity.

[44] Labrecque N., Cermakian N. (2015). Circadian Clocks in the Immune System. J. Biol. Rhythm..

[45] Bass J. (2012). Circadian topology of metabolism. Nat. Cell Biol..

[46] Bass J., Lazar M.A. (2016). Circadian time signatures of fitness and disease. Science.

[47] Pourcet B., Duez H. (2020). Circadian Control of Inflammasome Pathways: Implications for Circadian Medicine. Front. Immunol..

[48] LeGates T., Fernandez D.C., Hattar S. (2014). Light as a central modulator of circadian rhythms, sleep and affect. Nat. Rev. Neurosci..

[49] Dibner C., Schibler U., Albrecht U. (2010). The Mammalian Circadian Timing System: Organization and Coordination of Central and Peripheral Clocks. Annu. Rev. Physiol..

[50] Rijo-Ferreira F., Takahashi J.S. (2019). Genomics of circadian rhythms in health and disease. Genome Med..

[51] Sassone-Corsi P., Christen Y. (2016). The Epigenetic and Metabolic Language of the Circadian Clock. A Time for Metabolism and Hormones.

[52] Kim Y.H., Marhon S.A., Zhang Y., Steger D.J., Won K.-J., Lazar M.A. (2018). Rev-erbα dynamically modulates chromatin looping to control circadian gene transcription. Science.

[53] Reischl S., Kramer A. (2011). Kinases and phosphatases in the mammalian circadian clock. FEBS Lett..

[54] Hirano A., Yumimoto K., Tsunematsu R., Matsumoto M., Oyama M., Kozuka-Hata H., Nakagawa T., Lanjakornsiripan D., Nakayama K.I., Fukada Y. (2013). FBXL21 Regulates Oscillation of the Circadian Clock through Ubiquitination and Stabilization of Cryptochromes. Cell.

[55] Cardone L., Hirayama J., Giordano F., Tamaru T., Palvimo J.J., Sassone-Corsi P. (2005). Circadian Clock Control by SUMOylation of BMAL1. Science.

[56] Hirayama J., Sahar S., Grimaldi B., Tamaru T., Takamatsu K., Nakahata Y., Sassone-Corsi P. (2007). CLOCK-mediated acetylation of BMAL1 controls circadian function. Nat. Cell Biol..

[57] Hirano A., Fu Y.-H., Ptáček A.H.Y.-H.F.L.J. (2016). The intricate dance of post-translational modifications in the rhythm of life. Nat. Struct. Mol. Biol..

[58] Lazar M.A., Sassone-Corsi P., Christen Y. (2016). Rev-erbs: Integrating Metabolism Around the Clock. Stem Cells Neuroendocrinol..

[59] Ukai-Tadenuma M., Yamada R.G., Xu H., Ripperger J.A., Liu A.C., Ueda H.R. (2011). Delay in Feedback Repression by Cryptochrome 1 Is Required for Circadian Clock Function. Cell.

[60] Zhao X., Cho H., Yu R.T., Atkins A.R., Downes M., Evans R.M. (2014). Nuclear receptors rock around the clock. EMBO Rep..

[61] Teboul M., Guillaumond F., Gréchez-Cassiau A., Delaunay F. (2008). Minireview: The Nuclear Hormone Receptor Family Round the Clock. Mol. Endocrinol..

[62] Scheiermann C., Gibbs J., Ince L., Loudon A. (2018). Clocking in to immunity. Nat. Rev. Immunol..

[63] Balsalobre A., Brown S.A., Marcacci L., Tronche F., Kellendonk C., Reichardt H.M., Schütz G., Schibler U. (2000). Resetting of Circadian Time in Peripheral Tissues by Glucocorticoid Signaling. Science.

[64] So A.Y.-L., Bernal T.U., Pillsbury M.L., Yamamoto K.R., Feldman B.J. (2009). Glucocorticoid regulation of the circadian clock modulates glucose homeostasis. Proc. Natl. Acad. Sci. USA.

[65] Torra I.P., Tsibulsky V., Delaunay F., Saladin R., Laudet V., Fruchart J.-C., Kosykh V., Staels B. (2000). Circadian and glucocorticoid regulation of Rev-erbalpha expression in liver. Endocrinology.

[66] Lamia K.A., Papp S.J., Yu R.T., Barish G.D., Uhlenhaut H., Jonker J., Downes M., Evans R.M. (2011). Cryptochromes mediate rhythmic repression of the glucocorticoid receptor. Nat. Cell Biol..

[67] Nader N., Chrousos G.P., Kino T. (2009). Circadian rhythm transcription factor CLOCK regulates the transcriptional activity of the glucocorticoid receptor by acetylating its hinge region lysine cluster: Potential physiological implications. FASEB J..

[68] Nakamura T.J., Sellix M., Menaker M., Block G.D. (2008). Estrogen directly modulates circadian rhythms of PER2 expression in the uterus. Am. J. Physiol. Metab..

[69] Gery S., Virk R.K., Chumakov K., Yu A., Koeffler H.P. (2007). The clock gene Per2 links the circadian system to the estrogen receptor. Oncogene.

[70] Dufour C.R., Levasseur M.-P., Pham N.H.H., Eichner L.J., Wilson B.J., Charest-Marcotte A., Duguay D., Poirier-Héon J.-F., Cermakian N., Giguere V. (2011). Genomic Convergence among ERRα, PROX1, and BMAL1 in the Control of Metabolic Clock Outputs. PLoS Genet..

[71] Horard B., Rayet B., Triqueneaux G., Laudet V., Delaunay F., Vanacker J. (2004). Expression of the orphan nuclear receptor ERRalpha is under circadian regulation in estrogen-responsive tissues. J. Mol. Endocrinol..

[72] Gervois P., Chopin-Delannoy S., Fadel A., Dubois G., Kosykh V., Fruchart J.C., Najib J., Laudet V., Staels B. (1999). Fibrates increase human REV-ERBalpha ex-pression in liver via a novel peroxisome proliferator-activated receptor response element. Mol. Endocrinol..

[73] Fontaine C., Dubois G., Duguay Y., Helledie T., Vu-Dac N., Gervois P., Soncin F., Mandrup S., Fruchart J.C., Fruchart-Najib J. (2003). The orphan nuclear receptor Rev-Erbalpha is a pe-roxisome proliferator-activated receptor (PPAR) gamma target gene and promotes PPARgamma-induced adipocyte differentiation. J. Biol. Chem..

[74] Canaple L., Rambaud J., Dkhissi-Benyahya O., Rayet B., Tan N.S., Michalik L., Delaunay F., Wahli W., Laudet V. (2006). Reciprocal Regulation of Brain and Muscle Arnt-Like Protein 1 and Peroxisome Proliferator-Activated Receptor α Defines a Novel Positive Feedback Loop in the Rodent Liver Circadian Clock. Mol. Endocrinol..

[75] Wang N., Yang G., Jia Z., Zhang H., Aoyagi T., Soodvilai S., Symons J.D., Schnermann J.B., Gonzalez F.J., Litwin S.E. (2008). Vascular PPARgamma controls circadian variation in blood pressure and heart rate through Bmal1. Cell Metab..

[76] Pourcet B., Fruchart J.-C., Staels B., Glineur C. (2006). Selective PPAR modulators, dual and pan PPAR agonists: Multimodal drugs for the treatment of Type 2 diabetes and atherosclerosis. Expert Opin. Emerg. Drugs.

[77] McNamara P., Seo S.-B., Rudic D., Sehgal A., Chakravarti D., FitzGerald G.A. (2001). Regulation of CLOCK and MOP4 by Nuclear Hormone Receptors in the Vasculature: A Humoral Mechanism to Reset a Peripheral Clock. Cell.

[78] Rudic R.D., McNamara P., Reilly D., Grosser T., Curtis A.-M., Price T.S., Panda S., Hogenesch J.B., FitzGerald G.A. (2005). Bioinformatic Analysis of Circadian Gene Oscillation in Mouse Aorta. Circulation.

[79] Nonaka H., Emoto N., Ikeda K., Fukuya H., Rohman M.S., Raharjo S.B., Yagita K., Okamura H., Yokoyama M. (2001). Angiotensin II induces circadian gene expression of clock genes in cultured vascular smooth muscle cells. Circulation.

[80] Davidson A.J., London B., Block G.D., Menaker M. (2005). Cardiovascular tissues contain independent circadian clocks. Clin. Exp. Hypertens..

[81] Valentinuzzi V.S., Scarbrough K., Takahashi J.S., Turek F.W. (1997). Effects of aging on the circadian rhythm of wheel-running activity in C57BL/6 mice. Am. J. Physiol. Integr. Comp. Physiol..

[82] Chen C.-Y., Logan R.W., Ma T., Lewis D., Tseng G.C., Sibille E., McClung C.A. (2016). Effects of aging on circadian patterns of gene expression in the human prefrontal cortex. Proc. Natl. Acad. Sci. USA.

[83] Hood S., Amir S. (2017). The aging clock: Circadian rhythms and later life. J. Clin. Investig..

[84] Bubenik G.A., Konturek S.J. (2011). Melatonin and aging: Prospects for human treatment. J. Physiol. Pharmacol. Off. J. Pol. Physiol. Soc..

[85] Cuesta M., Boudreau P., Dubeau-Laramée G., Cermakian N., Boivin D.B. (2016). Simulated Night Shift Disrupts Circadian Rhythms of Immune Functions in Humans. J. Immunol..

[86] Wittmann M., Dinich J., Merrow M., Roenneberg T. (2006). Social Jetlag: Misalignment of Biological and Social Time. Chronobiol. Int..

[87] Wong P.M., Hasler B.P., Kamarck T.W., Muldoon M.F., Manuck S.B. (2015). Social Jetlag, Chronotype, and Cardiometabolic Risk. J. Clin. Endocrinol. Metab..

[88] Kantermann T., Duboutay F., Haubruge D., Kerkhofs M., Schmidt-Trucksäss A., Skene D.J. (2013). Atherosclerotic risk and social jetlag in rotating shift-workers: First evidence from a pilot study. Work.

[89] Sugiura T., Dohi Y., Takagi Y., Yoshikane N., Ito M., Suzuki K., Nagami T., Iwase M., Seo Y., Ohte N. (2019). Impacts of lifestyle behavior and shift work on visceral fat accumulation and the presence of atherosclerosis in middle-aged male workers. Hypertens. Res..

[90] Peñalvo J., Mertens E., Muñoz-Cabrejas A., León-Latre M., Jarauta E., Laclaustra M., Ordovás J., Casasnovas J., Uzhova I., Moreno-Franco B. (2021). Work Shift, Lifestyle Factors, and Subclinical Atherosclerosis in Spanish Male Workers: A Mediation Analysis. Nutrition.

[91] Bromfield S.G., Shimbo D., Booth J.N., Correa A., Ogedegbe G., Carson A.P., Muntner P. (2016). Cardiovascular Risk Factors and Masked Hyper-tension. Hypertension.

[92] Scheer F.A.J.L., Hilton M.F., Mantzoros C.S., Shea S.A. (2009). Adverse metabolic and cardiovascular consequences of circadian misalign-ment. Proc National Acad Sci. USA.

[93] Chellappa S., Vujovic N., Williams J.S., Scheer F.A. (2019). Impact of Circadian Disruption on Cardiovascular Function and Disease. Trends Endocrinol. Metab..

[94] McAlpine C.S., Kiss M.G., Rattik S., He S., Vassalli A., Valet C., Anzai A., Chan C.T., Mindur J.E., Kahles F. (2019). Sleep modulates haematopoiesis and protects against athero-sclerosis. Nature.

[95] Figueiro M.G., Goo Y., Hogan R., Plitnick B., Lee J., Jahangir K., Moulik M., Yechoor V.K., Paul A. (2020). Light-Dark Patterns Mirroring Shift Work Accelerate Athero-sclerosis and Promote Vulnerable Lesion Phenotypes. J. Am. Heart Assoc..

[96] Chalfant J.M., Howatt D.A., Tannock L.R., Daugherty A., Pendergast J.S. (2020). Circadian disruption with constant light exposure exac-erbates atherosclerosis in male ApolipoproteinE-deficient mice. Sci. Rep..

[97] Billon-Galés A., Krust A., Fontaine C., Abot A., Flouriot G., Toutain C., Berges H., Gadeau A.-P., Lenfant F., Gourdy P. (2011). Activation function 2 (AF2) of estrogen receptor-α is required for the atheroprotective action of estradiol but not to accelerate endothelial healing. Proc. Natl. Acad. Sci. USA.

[98] Billon-Galés A., Fontaine C., Douin-Echinard V., Delpy L., Berges H., Calippe B., Lenfant F., Laurell H., Guery J.-C., Gourdy P. (2009). Endothelial Estrogen Receptor-α Plays a Crucial Role in the Atheroprotective Action of 17β-Estradiol in Low-Density Lipoprotein Receptor–Deficient Mice. Circulation.

[99] Gourdy P., Guillaume M., Fontaine C., Adlanmerini M., Montagner A., Laurell H., Lenfant F., Arnal J.F. (2018). Estrogen receptor subcellular localization and cardiometabolism. Mol. Metab..

[100] Guivarc’H E., Buscato M., Guihot A., Favre J., Vessières E., Grimaud L., Wakim J., Melhem N., Zahreddine R., Adlanmerini M. (2018). Predominant Role of Nuclear Versus Membrane Estrogen Receptor α in Arterial Protection: Implications for Estrogen Receptor α Modulation in Cardiovascular Prevention/Safety. J. Am. Heart Assoc..

[101] Guillaume M., Montagner A., Fontaine C., Lenfant F., Arnal J.-F., Gourdy P. (2017). Sex and Gender Factors Affecting Metabolic Home-ostasis, Diabetes and Obesity. Adv. Exp. Med. Biol..

[102] Buscato M., Davezac M., Zahreddine R., Adlanmerini M., Métivier R., Fillet M., Cobraiville G., Moro C., Foidart J.-M., Lenfant F. (2021). Estetrol prevents Western diet–induced obesity and atheroma independently of hepatic estrogen receptor α. Am. J. Physiol. Metab..

[103] Zhu Z., Hua B., Shang Z., Yuan G., Xu L., Li E., Li X., Sun N., Yan Z., Qian R. (2016). Altered Clock and Lipid Metabolism-Related Genes in Atherosclerotic Mice Kept with Abnormal Lighting Condition. BioMed Res. Int..

[104] Schilperoort M., Berg R.V.D., Coomans C.P., Khedoe P.P.S.J., Ramkisoensing A., Boekestijn S., Wang Y., Berbée J.F.P., Meijer J.H., Biermasz N.R. (2020). Continuous Light Does Not Affect Atherosclerosis in APOE*3-Leiden.CETP Mice. J. Biol. Rhythm..

[105] Schilperoort M., Berg R.V.D., Bosmans L.A., Van Os B.W., Dollé M.E.T., Smits N., Guichelaar T., Van Baarle D., Koemans L., Berbée J.F.P. (2020). Disruption of circadian rhythm by alternating light-dark cycles aggravates atherosclerosis development in APOE*3-Leiden.CETP mice. J. Pineal Res..

[106] Cheng B., Anea C.B., Yao L., Chen F., Patel V., Merloiu A., Pati P., Caldwell R.W., Fulton D.J., Rudic R.D. (2011). Tissue-intrinsic dysfunction of circadian clock confers transplant arteriosclerosis. Proc. Natl. Acad. Sci. USA.

[107] Pan X., Bradfield C.A., Hussain M.M. (2016). Global and hepatocyte-specific ablation of Bmal1 induces hyperlipidaemia and enhances atherosclerosis. Nat. Commun..

[108] Pan X., Jiang X.-C., Hussain M.M. (2013). Impaired Cholesterol Metabolism and Enhanced Atherosclerosis in Clock Mutant Mice. Circulation.

[109] Yang L., Chu Y., Wang L., Wang Y., Zhao X., He W., Zhang P., Yang X., Liu X., Tian L. (2015). Overexpression of CRY1 protects against the development of athero-sclerosis via the TLR/NF-κB pathway. Int. Immunopharmacol..

[110] Mamontova A., Séguret-Macé S., Esposito B., Chaniale C., Bouly M., Delhaye-Bouchaud N., Luc G., Staels B., Duverger N., Mariani J. (1998). Severe Atherosclerosis and Hypoalphalipoproteinemia in the Staggerer Mouse, a Mutant of the Nuclear Receptor RORα. Circulation.

[111] Sitaula S., Billon C., Kamenecka T.M., Solt L.A., Burris T.P. (2015). Suppression of atherosclerosis by synthetic REV-ERB agonist. Biochem. Biophys. Res. Commun..

[112] Billon C., Sitaula S., Burris T.P. (2016). Inhibition of RORα/γ suppresses atherosclerosis via inhibition of both cholesterol absorption and inflammation. Mol. Metab..

[113] Anea C.B., Zhang M., Stepp D.W., Simkins G.B., Reed G., Fulton D.J., Rudic R.D. (2009). Vascular Disease in Mice With a Dysfunctional Circadian Clock. Circulation.

[114] Steffens S., Winter C., Schloss M.J., Hidalgo A., Weber C., Soehnlein O. (2017). Circadian Control of Inflammatory Processes in Athero-sclerosis and Its Complications. Arterioscler. Thromb. Vasc. Biol..

[115] McAlpine C.S., Swirski F. (2016). Circadian Influence on Metabolism and Inflammation in Atherosclerosis. Circ. Res..

[116] Turek F.W., Joshu C., Kohsaka A., Lin E., Ivanova G., McDearmon E., Laposky A., Losee-Olson S., Easton A., Jensen D.R. (2005). Obesity and Metabolic Syndrome in Circadian Clock Mutant Mice. Science.

[117] Narasimamurthy R., Hatori M., Nayak S.K., Liu F., Panda S., Verma I.M. (2012). Circadian clock protein cryptochrome regulates the expression of proinflammatory cytokines. Proc. Natl. Acad. Sci. USA.

[118] Winter C., Silvestre-Roig C., Ortega-Gomez A., Lemnitzer P., Poelman H., Schumski A., Winter J., Drechsler M., de Jong R., Immler R. (2018). Chrono-pharmacological Targeting of the CCL2-CCR2 Axis Ameliorates Atherosclerosis. Cell Metab..

[119] Ella K., Csépányi-Kömi R., Káldi K. (2016). Circadian regulation of human peripheral neutrophils. Brain, Behav. Immun..

[120] Casanova-Acebes M., Pitaval C., Weiss L.A., Nombela-Arrieta C., Chèvre R., Gonzalez N.A., Kunisaki Y., Zhang D., van Rooijen N., Silberstein L.E. (2013). Rhythmic Modulation of the Hematopoietic Niche through Neutrophil Clearance. Cell.

[121] Adrover J.M., del Fresno C., Crainiciuc G., Cuartero M., Casanova-Acebes M., Weiss L.A., Encabo H.H., Silvestre-Roig C., Rossaint J., Cossío I. (2019). A Neutrophil Timer Coordinates Immune Defense and Vascular Protection. Immunity.

[122] Schloss M.J., Horckmans M., Nitz K., Duchene J., Drechsler M., Bidzhekov K., Scheiermann C., Weber C., Soehnlein O., Steffens S. (2016). The time-of-day of myocardial infarction onset affects healing through oscillations in cardiac neutrophil recruitment. EMBO Mol. Med..

[123] Schloss M.J., Hilby M., Nitz K., Prats R.G., Ferraro B., Leoni G., Soehnlein O., Kessler T., He W., Luckow B. (2017). Ly6Chigh Monocytes Oscillate in the Heart During Homeostasis and After Myocardial Infarction-Brief Report. Arterioscler. Thromb. Vasc. Biology.

[124] Alibhai F.J., Tsimakouridze E.V., Chinnappareddy N., Wright D.C., Billia F., O’Sullivan M.L., Pyle W.G., Sole M.J., Martino T.A. (2014). Short-Term Disruption of Diurnal Rhythms After Murine Myocardial Infarction Adversely Affects Long-Term Myocardial Structure and Function. Circ. Res..

[125] Mistry P., Reitz C.J., Khatua T.N., Rasouli M., Oliphant K., Young M.E., Allen-Vercoe E., Martino T.A. (2020). Circadian influence on the microbiome improves heart failure outcomes. J. Mol. Cell. Cardiol..

[126] Tacke F., Alvarez D., Kaplan T.J., Jakubzick C., Spanbroek R., Llodra J., Garin A., Liu J., Mack M., van Rooijen N. (2007). Monocyte subsets differentially employ CCR2, CCR5, and CX3CR1 to accumulate within atherosclerotic plaques. J. Clin. Investig..

[127] Viswambharan H., Carvas J., Antic V., Marecic A., Jud C., Zaugg C.E., Ming X.-F., Montani J.-P., Albrecht U., Yang Z. (2007). Mutation of the Circadian Clock Gene Per2 Alters Vascular Endothelial Function. Circulation.

[128] Westgate E.J., Cheng Y., Reilly D.F., Price T.S., Walisser J.A., Bradfield C.A., FitzGerald G.A. (2008). Genetic Components of the Circadian Clock Regulate Thrombogenesis In Vivo. Circulation.

[129] Gao Y., Meng D., Sun N., Zhu Z., Zhao R., Lu C., Chen S., Hua L., Qian R. (2014). Clock upregulates intercellular adhesion molecule-1 expression and promotes mononuclear cells adhesion to endothelial cells. Biochem. Biophys. Res. Commun..

[130] Lutshumba J., Liu S., Zhong Y., Hou T., Daugherty A., Lu H., Guo Z., Gong M.C. (2018). Deletion of BMAL1 in Smooth Muscle Cells Protects Mice From Abdominal Aortic Aneurysms. Arter. Thromb. Vasc. Biol..

[131] Xie Z., Su W., Liu S., Zhao G., Esser K., Schroder E.A., Lefta M., Stauss H., Guo Z., Gong M.C. (2015). Smooth-muscle BMAL1 participates in blood pressure circadian rhythm regulation. J. Clin. Investig..

[132] Huo M., Huang Y., Qu D., Zhang H., Wong W.T., Chawla A., Huang Y., Tian X.Y. (2017). Myeloid Bmal1 deletion increases monocyte recruitment and worsens atherosclerosis. FASEB J..

[133] Yang G., Zhang J., Jiang T., Monslow J., Tang S.Y., Todd L., Puré E., Chen L., Fitzgerald G.A. (2020). Bmal1 Deletion in Myeloid Cells Attenuates Atherosclerotic Lesion Development and Restrains Abdominal Aortic Aneurysm Formation in Hyperlipidemic Mice. Arter. Thromb. Vasc. Biol..

[134] Lin Y., Tsai M., Hsieh I., Wen M., Wang C. (2021). Deficiency of circadian gene cryptochromes in bone marrow-derived cells protects against atherosclerosis in LDLR^−/−^ mice. FASEB J..

[135] Ma H., Zhong W., Jiang Y., Fontaine C., Li S., Fu J., Olkkonen V.M., Staels B., Yan D. (2013). Increased atherosclerotic lesions in LDL receptor deficient mice with hematopoietic nuclear receptor Rev-erbalpha knock-down. J. Am. Heart Assoc..

[136] Liang S., Hu J., Zhang A., Li F., Li X. (2020). miR-155 induces endothelial cell apoptosis and inflammatory response in atherosclerosis by regulating Bmal1. Exp. Ther. Med..

[137] Schober A., Blay R.M., Maleki S.S., Zahedi F., Winklmaier A.E., Kakar M.Y., Baatsch I.M., Zhu M., Geißler C., Fusco A.E. (2021). MicroRNA-21 Controls Circadian Regulation of Apoptosis in Atherosclerotic Lesions. Circulation.

[138] Kurakula K., Hamers A.A.J., de Waard V., de Vries C.J.M. (2013). Nuclear Receptors in atherosclerosis: A superfamily with many ‘Good-fellas’. Mol. Cell Endocrinol..

[139] Neels J.G., Hassen-Khodja R., Chinetti G. (2020). Nuclear receptors in abdominal aortic aneurysms. Atherosclerosis.

[140] Duez H., Pourcet B. (2021). Nuclear Receptors in the Control of the NLRP3 Inflammasome Pathway. Front. Endocrinol..

[141] Fontaine C., Rigamonti E., Pourcet B., Duez H., Duhem C., Fruchart J.C., Chinetti-Gbaguidi G., Staels B. (2008). The nuclear receptor Rev-erbalpha is a liver X receptor (LXR) target gene driving a negative feedback loop on select LXR-induced pathways in human macrophages. Mol. Endocrinol..

[142] Yin L., Wu N., Curtin J.C., Qatanani M., Szwergold N.R., Reid R.A., Waitt G.M., Parks D.J., Pearce K.H., Wisely G.B. (2007). Rev-erb, a Heme Sensor That Coordinates Metabolic and Circadian Pathways. Science.

[143] Dierickx P., Emmett M.J., Jiang C., Uehara K., Liu M., Adlanmerini M., Lazar M.A. (2019). SR9009 has REV-ERB–independent effects on cell proliferation and metabolism. Proc. Natl. Acad. Sci. USA.

[144] Trump R.P., Bresciani S., Cooper A.W., Tellam J.P., Wojno J., Blaikley J., Orband-Miller L.A., Kashatus J.A., Boudjelal M., Dawson H.C. (2013). Optimized chemical probes for REV-ERBalpha. J. Med. Chem..

[145] Banerjee S., Wang Y., Solt L.A., Griffett K., Kazantzis M., Amador A., El-Gendy B.M., Huitron-Resendiz S., Roberts A.J., Shin Y. (2014). Pharmacological targeting of the mammalian clock regulates sleep architecture and emotional behaviour. Nat. Commun..

[146] Pariollaud M., Gibbs J., Hopwood T., Brown S., Begley N., Vonslow R., Poolman T., Guo B., Saer B., Jones D.H. (2018). Circadian clock component REV-ERBalpha controls homeostatic regulation of pulmonary inflammation. J. Clin. Investig..

[147] Marciano D.P., Chang M.R., Corzo C.A., Goswami D., Lam V.Q., Pascal B.D., Griffin P.R. (2014). The Therapeutic Potential of Nuclear Receptor Modulators for Treatment of Metabolic Disorders: PPARγ, RORs, and Rev-erbs. Cell Metab..

[148] Duez H., Staels B. (2009). Rev-erb-α: An integrator of circadian rhythms and metabolism. J. Appl. Physiol..

[149] Kojetin D.J., Burris T.P. (2014). REV-ERB and ROR nuclear receptors as drug targets. Nat. Rev. Drug Discov..

[150] Duez H., van der Veen J.N., Duhem C., Pourcet B., Touvier T., Fontaine C., Derudas B., Baugé E., Havinga R., Bloks V.W. (2008). Regulation of bile acid synthesis by the nuclear receptor Rev-erbalpha. Gastroenterology.

[151] Vu-Dac N., Gervois P., Grotzinger T., Vos P.D., Schoonjans K., Fruchart J.C., Auwerx J., Mariani J., Tedgui A., Staels B. (1997). Transcriptional regulation of apolipoprotein A-I gene expression by the nuclear receptor RORalpha. J. Biol. Chem..

[152] Raspe E., Duez H., Mansen A., Fontaine C., Fievet C., Fruchart J.C., Vennström B., Staels B. (2002). Identification of Rev-erbalpha as a physiological repressor of apoC-III gene transcription. J. Lipid Res..

[153] Coste H., Rodríguez J.C. (2002). Orphan Nuclear Hormone Receptor Rev-erbα Regulates the Human Apolipoprotein CIII Promoter. J. Biol. Chem..

[154] Jia X., Rifai M.A., Hussain A., Martin S., Agarwala A., Virani S.S. (2020). Highlights from Studies in Cardiovascular Disease Prevention Presented at the Digital 2020 European Society of Cardiology Congress: Prevention Is Alive and Well. Curr. Atheroscler. Rep..

[155] Raspe E., Duez H., Gervois P., Fievet C., Fruchart J.C., Besnard S., Mariani J., Tedgui A., Staels B. (2001). Transcriptional regulation of apolipoprotein C-III gene expression by the orphan nuclear receptor RORalpha. J. Biol. Chem..

[156] Gibbs J.E., Blaikley J., Beesley S., Matthews L., Simpson K.D., Boyce S.H., Farrow S.N., Else K.J., Singh D., Ray D.W. (2012). The nuclear receptor REV-ERBα mediates circadian regulation of innate immunity through selective regulation of inflammatory cytokines. Proc. Natl. Acad. Sci. USA.

[157] Pourcet B., Zecchin M., Ferri L., Beauchamp J., Sitaula S., Billon C., Delhaye S., Vanhoutte J., Mayeuf-Louchart A., Thorel Q. (2018). Nuclear Receptor Subfamily 1 Group D Member 1 Reg-ulates Circadian Activity of NLRP3 Inflammasome to Reduce the Severity of Fulminant Hepatitis in Mice. Gastroenterology.

[158] Delerive P., Monte D., Dubois G., Trottein F., Fruchart-Najib J., Mariani J., Fruchart J., Staels B. (2001). The orphan nuclear receptor RORα is a negative regulator of the inflammatory response. EMBO Rep..

[159] Billon C., Murray M.H., Avdagic A., Burris T.P. (2019). RORγ regulates the NLRP3 inflammasome. J. Biol. Chem..

[160] Duewell P., Kono H., Rayner K.J., Sirois C.M., Vladimer G., Bauernfeind F.G., Abela G.S., Franchi L., Nuñez G., Schnurr M. (2010). NLRP3 inflammasomes are required for ather-ogenesis and activated by cholesterol crystals. Nature.

[161] van der Heijden T., Kritikou E., Venema W., van Duijn J., van Santbrink P.J., Slütter B., Foks A.C., Bot I., Kuiper J. (2017). NLRP3 Inflammasome Inhibition by MCC950 Reduces Atherosclerotic Lesion Development in Apolipoprotein E-Deficient Mice-Brief Report. Arterioscler. Thromb. Vasc. Biology.

[162] Sheedy F., Grebe A., Rayner K., Kalantari P., Ramkhelawon B., Carpenter S.B., Becker C.E., Ediriweera H.N., Mullick A., Golenbock D.T. (2013). CD36 coordinates NLRP3 inflammasome activation by facilitating intracellular nucleation of soluble ligands into particulate ligands in sterile inflammation. Nat. Immunol..

[163] Stewart C.R., Stuart L.M., Lacy-Hulbert A., El Khoury J., Golenbock D.T., Moore K.J., Wilkinson K., van Gils J.M., Deng J., Halle A. (2010). CD36 ligands promote sterile inflammation through assembly of a Toll-like receptor 4 and 6 heterodimer. Nat. Immunol..

[164] Wang S., Lin Y., Yuan X., Li F., Guo L., Wu B. (2018). REV-ERBα integrates colon clock with experimental colitis through regulation of NF-κB/NLRP3 axis. Nat. Commun..

[165] Yu D., Fang X., Xu Y., Xiao H., Huang T., Zhang Y., Ge Y., Li Y., Zong L., Gao J. (2019). Rev-erbα can regulate the NF-κB/NALP3 pathway to modulate lipo-polysaccharide-induced acute lung injury and inflammation. Int. Immunopharmacol..

[166] Woldt E., Sebti Y., Solt L.A., Duhem C., Lancel S., Eeckhoute J., Hesselink M.K.C., Paquet C., Delhaye S., Shin Y. (2013). Rev-erb-α modulates skeletal muscle oxidative capacity by regulating mitochondrial biogenesis and autophagy. Nat. Med..

[167] Lin Y., Wang S., Gao L., Zhou Z., Yang Z., Lin J., Ren S., Xing H., Wu B. (2021). Oscillating lncRNA Platr4 regulates NLRP3 inflammasome to ameliorate nonalcoholic steatohepatitis in mice. Theranostics.

[168] Wang S., Lin Y., Li F., Qin Z., Zhou Z., Gao L., Yang Z., Wang Z., Wu B. (2020). An NF-κB–driven lncRNA orchestrates colitis and circadian clock. Sci. Adv..

[169] Sulli G., Manoogian E.N., Taub P.R., Panda S. (2018). Training the Circadian Clock, Clocking the Drugs, and Drugging the Clock to Prevent, Manage, and Treat Chronic Diseases. Trends Pharmacol. Sci..

